# Systemic inflammation and eye diseases. The Beijing Eye Study

**DOI:** 10.1371/journal.pone.0204263

**Published:** 2018-10-03

**Authors:** Jost B. Jonas, Wen Bin Wei, Liang Xu, Ya Xing Wang

**Affiliations:** 1 Beijing Institute of Ophthalmology, Beijing Key Laboratory of Ophthalmology and Visual Sciences, Beijing Tongren Eye Center, Beijing Tongren Hospital, Capital Medical University; Beijing, China; 2 Department of Ophthalmology, Medical Faculty Mannheim of the Ruprecht-Karls-University, Mannheim, Germany; 3 Beijing Tongren Eye Center, Beijing key Laboratory of Intraocular Tumor Diagnosis and Treatment, Beijing Tongren Hospital, Capital Medical University, Beijing, China; University of Tasmania, AUSTRALIA

## Abstract

**Purpose:**

Systemic inflammation is potentially associated with ocular diseases such as late age-related macular degeneration (AMD). Using the serum concentration of high-sensitive C-reactive protein (hs-CRP) as surrogate of systemic inflammation, we examined potential associations between the serum hs-CRP concentration and the presence and degree of eye diseases.

**Methods:**

The population-based Beijing Eye Study included 3468 Chinese individuals. The study participants underwent a standardized interview and a detailed ophthalmic examination. The serum concentration of hs-CRP was determined.

**Results:**

Out of 3468 participants, 2452 (70.7%) individuals (mean age:63.4±9.4 year; range:50–91 years) had hs-CRP measurements (mean:1.96±4.07mg/L). In multivariate analysis, higher serum concentration of hs-CRP was significantly (regression coefficient r: 0.21) associated with a higher level of diabetic retinopathy (*P* = 0.007; standardized regression coefficient beta:0.06; non-standardized regression coefficient B:1.35; 95% confidence interval (CI):0.37,2.22) and polypoidal choroidal vasculopathy (*P* = 0.002;beta:0.06;B:6.22;95%CI:2.24,10.2) after adjusting for higher serum concentration of high-density lipoproteins (*P*<0.001;beta:-0.12;B:-1.31;95%CI:-1.77,-0.85), higher body mass index (*P* = 0.01;beta:0.06;B:0.06;95%CI:0.01, 0.11), lower level of education (*P* = 0.04;beta:-0.06;B:-0.22;95%CI:-0.42,-0.02), lower cognitive function score (*P* = 0.01;beta:-0.07;B:-0.08;95%CI:-0.13,-0.02). If the presences of other ocular diseases were added to the model, the presence of glaucoma (*P* = 0.99), open-angle glaucoma (*P* = 0.80), angle-closure glaucoma (*P* = 0.67), pseudoexfoliation (*P* = 0.18), nuclear cataract (*P* = 0.30), cortical cataract (*P* = 0.15), subcapsular cataract (*P* = 0.59), retinal vein occlusions (*P* = 0.33), central serous choroidopathy (*P* = 0.44), early stage of age-related macular degeneration (AMD) (*P* = 0.46), intermediate stage of AMD (*P* = 0.20) and late stage of AMD (*P* = 0.91) including geographic atrophy (*P* = 0.60) or neovascular AMD (*P* = 0.68) were not significantly associated with the serum concentration of hs-CRP.

**Conclusions:**

In Chinese aged 50+ years, higher serum concentration of hs-CRP was significantly associated with a higher level of diabetic retinopathy and higher frequency of polypoidal choroidal vasculopathy. Other major ocular disorders, namely glaucoma including open-angle glaucoma and angle-closure glaucoma, pseudoexfoliation, nuclear, cortical or subcapsular cataract, retinal vein occlusions, central serous choroidopathy, early, intermediate or late stage of AMD including geographic atrophy, were not significantly associated with hs-CRP serum concentrations. It suggests that these diseases, in contrast to diabetic retinopathy and polypoidal choroidal vasculopathy, were not associated with a major systemic inflammatory component.

## Introduction

Inflammation is an important parameter in major disorders and has been implicated in the pathogenesis of ocular diseases such as age-related macular degeneration (AMD) and diabetic retinopathy [1–6]. These diseases are associated with typical features of inflammation such as increased vessel wall permeability, leakage into the surrounding interstitial tissue and edema, activation of blood-borne inflammatory cells such, and involvement of the complement system, to cite only few inflammation-associated changes. Few clinical studies have so far examined the component of a systemic subclinical inflammation in association with eye diseases, and if performed, these studies were concentrated on AMD [7–17]. We therefore conducted this investigation to assess whether the level of systemic inflammation, measured by the serum concentration of C-reactive protein (CRP), is associated with the occurrence of eye diseases, including AMD and other major ocular disorders. CRP is a ring-shaped pentameric protein which is synthesized by the liver and secreted into the blood in response to inflammation, following interleukin-6 secretion by macrophages and T-cells. CRP binds to lysophosphatidylcholine expressed on the surface of dead or dying cells, in order to activate the complement system. CRP is a biomarker of systemic inflammation as shown in numerous previous investigations [18,19]. To reduce the risk of a referral bias, we chose a population-based recruitment of the study participants.

## Methods

The Beijing Eye Study was a population-based study performed in a rural region and in an urban region of Greater Beijing. Its protocol was approved by the Medical Ethics Committee of Beijing Tongren Hospital, and all study participants gave an informed consent. The study was conducted in four communities in the urban district of Haidian in the North of Central Beijing, and in three communities in the rural area of Yufa of the Daxing District South of Beijing. Out of 4403 eligible individuals with an age of 50+ years, 3468 subjects (1963 (56.6%) women) with a mean age of 64.6 ± 9.8 years (median 64 years; range: 50–93 years) participated (response rate: 78.8%). The study has been described in detail previously [20,21].

The study procedures included an interview carried out by trained health personal and including standardized questions on the socioeconomic background, depression, physical activity, known major systemic diseases such as arterial hypertension and diabetes mellitus, intake of systemic medication, smoking habits and alcoholic consumption, measurement of serum concentrations of lipids, glucose, glycosylated hemoglobin HbA1c and CRP in blood samples collected under fasting conditions, determination of blood pressure, body height, body weight and circumference of the waist and hip, and ophthalmological examinations consisting of assessment of best corrected visual acuity, tonometry, slit lamp assisted biomicroscopy of the anterior and posterior segment of the eyes, digital photography of the cornea and lens, ocular biometry (of the right eyes) using optical low-coherence reflectometry, spectral-domain optical coherence tomography of the macula and optic nerve head, and fundus color photography (45°; fundus camera CR6-45NM, Canon Inc. U.S.A.) centered on the optic disc and macula. The CRP concentration was measured as high sensitive CRP (hs-CRP), using the CRP kit Roche (Roche Co, Basel/Kaiseraugst, Switzerland). The measurement unit was mg/L.

The level of education was categorized into various degrees. “Illiteracy” was considered as the inability to read any Chinese word. “Half illiteracy” was present when the person could read some Chinese words, but could not obtain any useful information from the reading. The next degrees of literacy were “Primary School,” “Middle School,” and “College Education,” respectively, if they had been attended. Self-reported income was differentiated into the categories of an income of less than 500 Yuan, 501 to 1000 Yuan, 1001 to 2000 Yuan, 2001 to 3000 Yuan, 3001 to 5000 Yuan, 5001 to 8000 Yuan, 8001 to 10000 Yuan, and more than 10000 Yuan. The cognitive function was assessed using the MMSE (mini mental state examination) scale [22]. The frequency of alcohol consumption was categorized as “less than once per month”, “once per month”, twice to thrice per month”, “once per week”, “twice to thrice per week”, and “every day”.

The degree of cataract was determined using the lens photographs. The degree of nuclear opacities was assessed in 6 grades using the grading system of the Age-Related Eye Disease Study [23]. In addition, retro-illuminated photographs of the lens were obtained (Neitz CT-R camera; Neitz Instruments Co., Tokyo, Japan). On these photographs, the cortical and posterior subcapsular opacities appeared as darkly shaded areas on a white background. The percentage of the areas with lens opacities was measured using a grid. The standard to diagnose a nuclear cataract was a nuclear cataract grade of 4 or more, the standard to diagnose a posterior subcapsular cataract was an amount of posterior subcapsular opacities of 0.01 or more, and the standard to diagnose a cortical cataract was an amount of cortical opacities of 0.05 or more [23]. The definition of glaucoma was based on the examination of photographs of the optic disc and macula. The optic nerve head was glaucomatous (1) if the inferior-superior-nasal-temporal (ISNT)-rule of the neuroretinal rim shape was not fulfilled in early glaucoma and in eyes with a normally shaped optic disc (it included a notch in the neuroretinal rim in the temporal inferior region and / or the temporal superior region); or (2) if an abnormally large cup was present in a small optic disc which normally would not show cupping [24]. Additionally, glaucomatous optic neuropathy was defined according to the criteria of the International Society of Geographic and Epidemiological Ophthalmology ISGEO [24,25]. Pseudoexfoliation was assessed by an experienced ophthalmologist during the slit lamp assisted biomicroscopy of the anterior segment after pupillary dilation. The diagnosis of pseudoexfoliation was definite, if the lens surface showed a central whitish coating with a diameter of little less than the normal pupillary diameter, or if the periphery of the lens surface showed a whitish coating which was anteriorly bordered by a darker ring-like region on the lens surface. The assessment of pseudoexfoliation was performed only in phakic eyes. Retinal vein occlusions were differentiated into central retinal vein occlusions characterized by retinal edema, optic disc hyperemia or edema, scattered superficial or deep retinal hemorrhages and venous dilation in its acute stage, and by occluded and sheathed retinal veins or vascular anastomoses at the optic disc in the chronic stage; and into branch retinal vein occlusions showing localized retinal edema, superficial and deep retinal hemorrhages, intraretinal microvascular abnormalities or anastomotic vessels, and venous dilatation or venous sheathing within a sector of the retina corresponding to the obstructed vein. Diabetic retinopathy was assessed on the fundus photographs using the Early Treatment of Diabetic Retinopathy Study (ETDRS) criteria. Using the international Clinical Diabetic Retinopathy scale, diabetic retinopathy was graded into the stages of mild non-proliferative diabetic retinopathy, moderate non-proliferative diabetic retinopathy, severe non-proliferative diabetic retinopathy, and proliferative diabetic retinopathy. The minimum criterion for diagnosis of diabetic retinopathy was the presence of at least one microaneurysm or dot-like hemorrhage and the diagnosis of diabetes mellitus. The diagnosis for each individual was based on the grading of the individual´s eye with the highest stage of diabetic retinopathy. Central serous choroidopathy was defined as a serous retinal detachment without hemorrhage and without marked drusen both on fundus photographs and OCT images. For the diagnosis of AMD, the International ARM (Age-related Maculopathy Epidemiological Study Group) Grading system was used [23]. Polypoidal choroidal vasculopathy was defined as an elevated orange-red lesion on the fundus photographs and was characterized by a double-layer sign and high dome-shaped pigment epithelial detachments on the OCT images. The degree of fundus tessellation defined as the visibility of the large choroidal vessels was assessed on the 45° fundus photographs of the macula and of the optic disc as described in detail previously [26]. Fundus tessellation was differentiated between grade “0” for “no tessellation” and grade”3” for “marked tessellation”.

Arterial hypertension was defined by systolic blood pressure ≥160 mmHg, diastolic blood pressure ≥95 mmHg, or by previous history of hypertension or use of antihypertensive medication. The diagnosis of diabetes mellitus was based on serum concentration of glucose ≥7 mmol/L, or a concentration of glycosylated hemoglobin HbA1c of ≥6%, or a history of diabetes mellitus including diabetes therapy.

Statistical analysis was performed using a commercially available statistical software package (SPSS for Windows, version 22.0, IBM-SPSS, Chicago, IL). In a first step, we calculated the mean concentration of hs-CRP (mean ± standard deviation). We then assessed associations between the hs-CRP measurements and systemic and ocular parameters in univariate analysis. We then conducted a multivariate linear regression analysis with the hs-CRP measurements as dependent variable and as independent variables all those parameters which were significantly associated with hs-CRP concentrations in the univariate analysis. Finally, we assessed in a binary regression analysis associations of the parameter of an hs-CRP serum concentration of higher than 3mg/L. We choose the cut-off value of 3mg/L which corresponds to the approximate borderline between the medium tertile and upper tertile of the hs-CRP distribution in the adult population and which has been considered to be the upper limit of the average hs-CRP-defined risk for coronary artery diseases [27]. We calculated the standardized regression coefficient beta, the non-standardized regression coefficient B, the odds ratio (OR) and the 95% confidence intervals (CI). A *P*-value <0.05 was considered to indicate statistical significance.

## Results

Out of 3468 participants of the Beijing Eye Study 2011, measurements of hs-CRP were available for 2452 (70.7%) individuals (1032 (42.1%) men) with a mean age of 63.4 ± 9.4 years (range: 50–91 years). Out of the 2452 participants, 1422 (58.0%) individuals came from the rural region and 1030 (42.0%) participants lived in the urban region. The group of individuals participating compared to the group of individuals without measurements of hs-CRP was significantly younger (63.4 ± 9.4 years versus 67.4 ± 10.2 years; *P*<0.001), came significantly more often from the urban region than from the rural area (rural / urban region of habitation: 1422 / 1030 versus 204 / 800; *P*<0.001), had a significantly shorter axial length (23.1 ± 1.1 mm versus 23.6 ± 1.2 mm; *P*<0.001) and was significantly less myopic (-0.13 ± 1.97 diopters versus -0.47 ± 2.46 diopters; *P*<0.001). They did not differ significantly in proportion of women versus men (men / women: 1033 / 1419 versus 466 / 538; *P* = 0.21).

The mild non-proliferative stage of diabetic retinopathy was present in 39 (1.6%) individuals, the moderate non-proliferative stage of diabetic retinopathy was present in 25 (1.0%) individuals, the severe non-proliferative stage of diabetic retinopathy was present in 11 (0.4%) individuals, and the proliferative stage of diabetic retinopathy was present in 10 (0.4%) individuals. The mean hs-CRP concentration was 1.96 ± 4.07 mg/L (median 0.95 mg/L). A value higher than 3 mg/l was found in 378 (15.4%; 95%CI: 14.0, 16.9) of the study participants.

**Fig 1:**
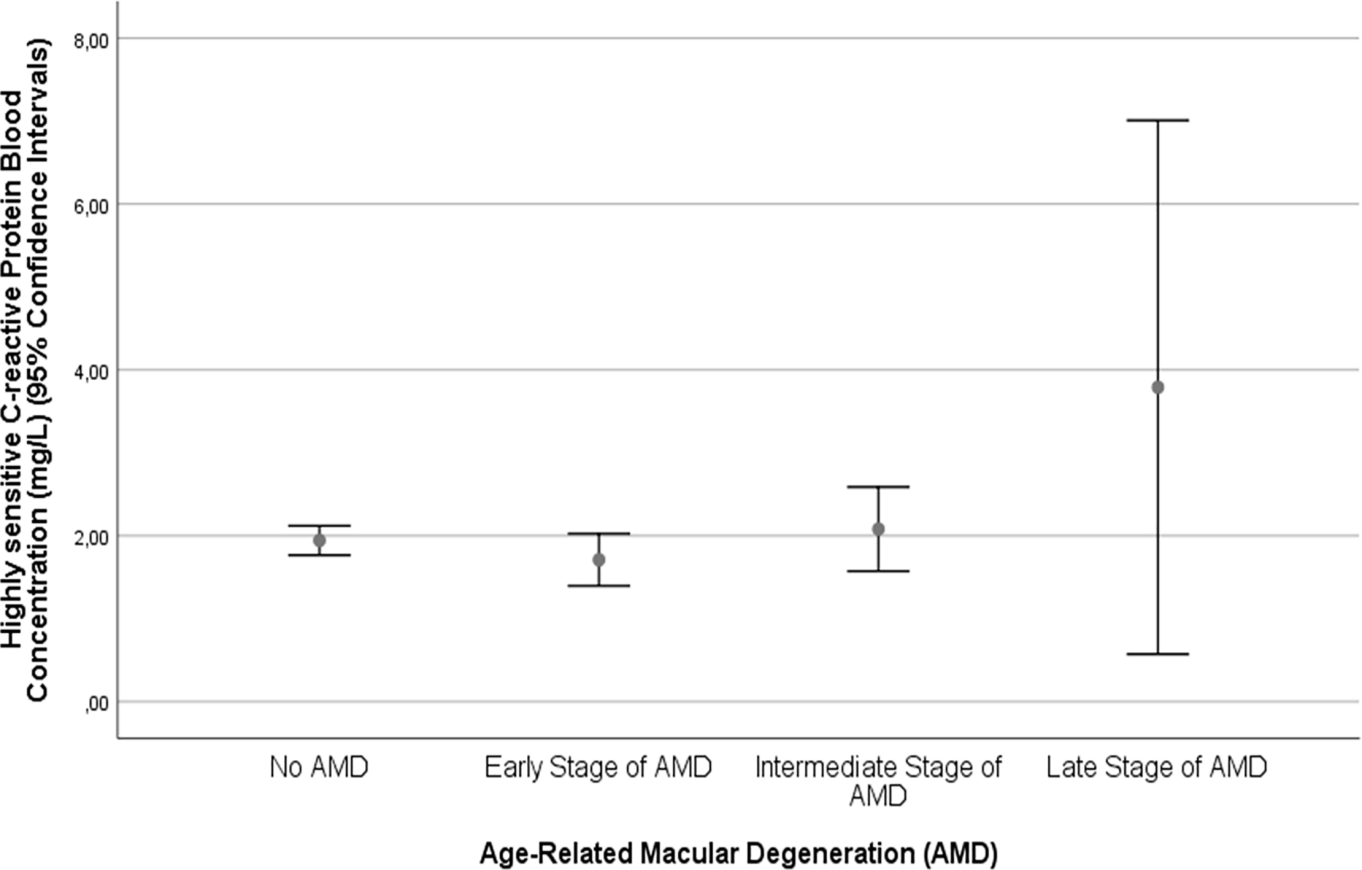
Graph showing the distribution of hs-C-reactive protein in the Beijing Eye Study, stratified by the stage of age-related macular degeneration.

**Fig 2:**
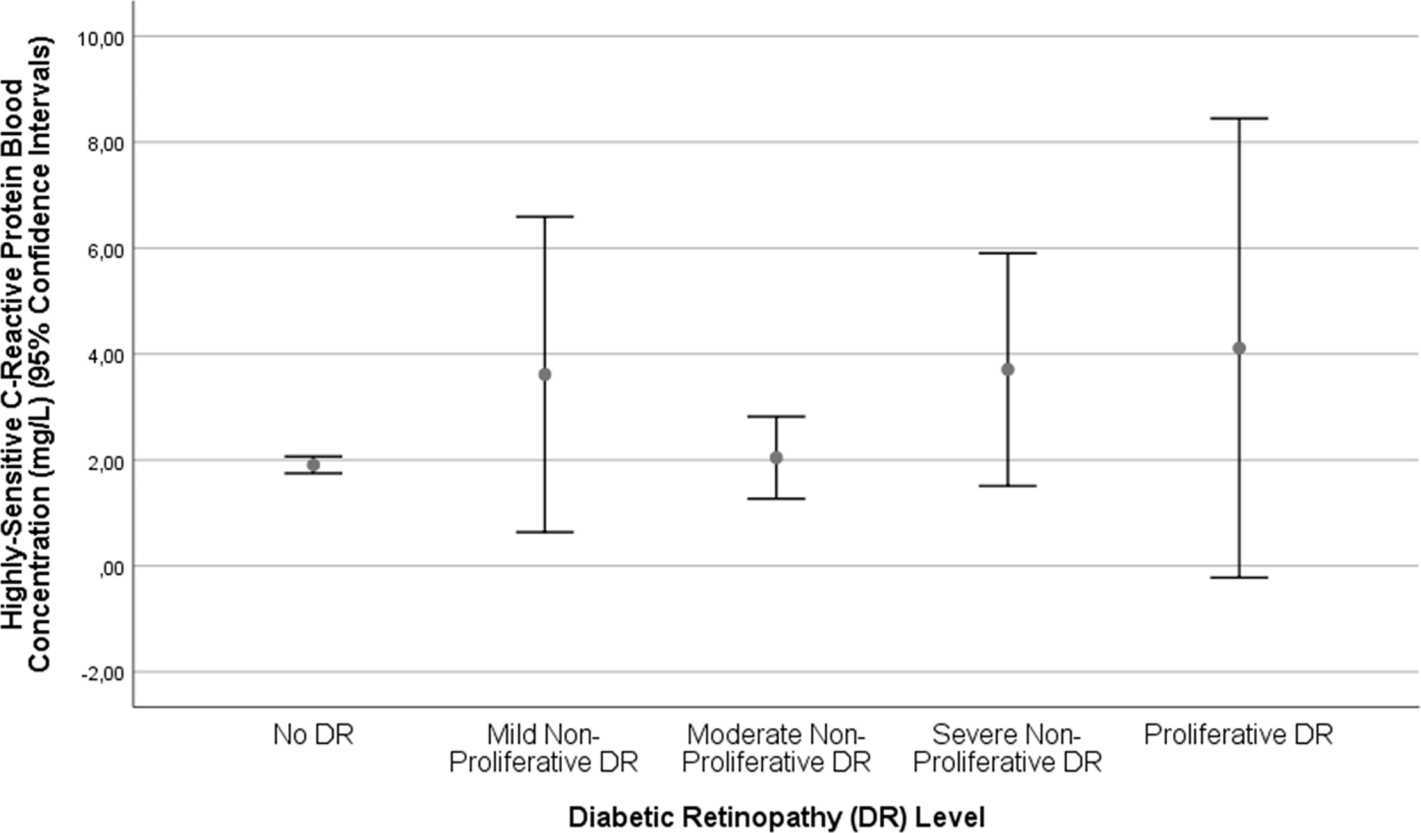
Graph showing the distribution of hs-C-reactive protein in the Beijing Eye Study, stratified by the stage of diabetic retinopathy.

In univariate analysis, higher serum concentration of hs-CRP was associated (*P*≤0.10) with the systemic parameters of older age (*P* = 0.013), rural region of habitation (*P* = 0.002), higher body mass index (*P*<0.001), lower level of education (*P*<0.001), lower self-reported income (*P*<0.001), lower cognitive score (*P*<0.001), lower frequency of alcohol consumption (*P* = 0.003), higher serum concentration of glucose (*P*<0.001), glycosylated hemoglobin (*P*<0.001) and lower serum concentrations of high-density lipoproteins (*P*<0.001), higher frequency of diabetes mellitus (*P*<0.001), and higher systolic blood pressure (*P =* 0.04), and intake of aspirin (*P* = 0.05). It was associated with the ocular parameters of shorter axial length (*P* = 0.09), higher degree of nuclear cataract (*P* = 0.04), higher frequency of cortical cataract (*P* = 0.05) and subcapsular posterior cataract (*P* = 0.07), neovascular stage of neovascular AMD (*P* = 0.04) (Fig 1), diabetic retinopathy (*P* = 0.003) (Fig 2), and polypoidal choroidal vasculopathy (*P* = 0.002) (Table 1). Within the group of patients with diabetes mellitus (n = 385), the hs-CRP concentration was higher, however not statistically significantly, in the subgroup with diabetic retinopathy (n = 74) than in the subgroup without diabetic retinopathy (n = 311) (3.51 ± 7.95 mg/L versus 2.18 ± 2.93 mg/L; *P* = 0.13).

**Table 1:** Associations (univariate analysis) between the serum concentration of hs-C-reactive protein and other systemic and ocular parameters in the Beijing Eye Study.

Parameter	Mean ± Standard Deviation / Frequency (95% Confidence Intervals)	*P*-Value	Standardized Regression Coefficient beta	Non-Standardized Regression Coefficient B	95% Confidence Interval
Age (Years)	63.4 ± 9.4	0.01	0.05	0.02	0.01, 0.04
Gender (Men / Women)	1032 / 1420	0.21			
Rural / Urban Region of Habitation	1422 / 1030	0.002	-0.06	-0.52	-0.84, -0.19
Body Mass Index (kg/m^2^)	25.8 ± 3.9	<0.001	0.11	0.11	0.07, 0.15
Level of Education (Levels 1–5)	3.8 ± 1.1	<0.001	-0.10	-0.39	-0.55, -0.23
Self-Reported Income (Categories: <500 Yuan; -1000; -2000; -3000; -5000; -8000; -10000; >10000)	3.9 ± 2.2	<0.001	-0.07	-0.14	-0.21, -0.06
Cognitive Score	26.2 ± 3.7	<0.001	-0.10	-0.11	-0.16, -0.07
Alcohol Consumption Frequency	15.6% (14.0, 17.3)	0.02	-0.05	-0.11	-0.20, -0.02
Smoking (Never / Former/ Current)	1671 / 233 / 548	0.57			
Smoking (Never / Ever)	1671 / 781	0.48			
Smoking Package Years	10.5 ± 19.6	0.13			
Physical Activity					
Number of Hours Spent With Vigorously Intensive Physical Activities Per Week	0.8 ± 5.9	0.13			
Number of Hours Spent With Moderate Physical Activities Per week	11.7 ± 21.0	0.04	-0.04	-0.06	-0.11, -0.002
Number of Hours Spent With Sitting Per Week	35.3 ± 17.5	0.34			
Number of Hours Spent With Walking Per Week	16.9 ± 14.5	0.33			
Blood Concentration of:					
Glucose (mmol/L)	5.72 ± 1.53	<0.001	0.08	0.21	0.10, 0.31
Glycosylated Hemoglobin HbA1c (%)	4.36 ± 1.04	<0.001	0.10	0.38	0.23, 0.54
High-Density Lipoproteins (mmol/L)	1.43 ± 0.39	<0.001	-0.14	-1.47	-1.88, -1.06
Low-Density Lipoproteins (mmol/L)	3.37 ± 0.87	0.86			
Triglycerides (mmol/L)	1.62 ± 1.17	0.08			
Cholesterol (mmol/L)	5.08 ± 0.96	0.14			
Diabetes Mellitus, Frequency	15.7% (14.3, 17.1)	0.02	0.05	0.56	0.11, 1.00
Systolic Blood Pressure (mmHg)	131.7 ± 20.8	0.04	0.04	0.01	0, 0.02
Diastolic Blood Pressure (mmHg)	71.3 ± 12.4	0.78			
Mean Blood Pressure (mmHg)	91.4 ± 14.1	0.25			
Arterial Hypertension, Frequency	45.2 (43.2, 47.2)	0.11			
Estimated Glomerular Filtration Rate (mL/min / 1·73 m^2^) (CKDE Formula; China Adapted)	90.6 ± 12.1	0.50			
Chronic Kidney Disease, Frequency (GFR<60 mL/min / 1·73 m^2^) (CKDE Formula; China Adapted), Frequency	2.2% (1.7, 2.8)	0.39			
Use of aspirin, Frequency	29.8% (27.9, 31.6)	0.05	0.04	0.36	-0.005, 0.72
Depression Score	22.7 ± 5.3	0.48			
Ophthalmological Parameters					
Axial Length (mm)	23.1 ± 1.1	0.09	-0.04	-0.13	-0.29, 0.02
Anterior Corneal Curvature Radius (mm)	7.60 ± 0.25	0.18			
Central Corneal Thickness (μm)	531 ± 33	0.26			
Anterior Chamber Depth (mm)	2.47 ± 0.46	0.44			
Lens Thickness (mm)	4.56 ± 0.33	0.81			
Intraocular Pressure mmHg)	14.6 ± 2.7	0.81			
Retinal Nerve Fiber Layer Thickness (μm)	101 ± 12	0.37			
Subfoveal Choroidal Thickness (μm)	261 ± 106	0.18			
Fundus Tessellation (Grades 0–3)	0.7 ± 0.7	0.82			
Macular Retinal Thickness (μm)	243 ± 35	0.11			
Optic Disc Size (mm^2^)	1.90 ± 0.42	0.19			
Parapapillary Alpha Zone Area (mm^2^)	0.48 ± 0.55	0.82			
Parapapillary Beta Zone Area (mm^2^)	0.27 ± 1.00	0.57			
Parapapillary Gamma Zone Width (mm)	0.14 ± 0.59	0.47			
Nuclear Cataract, Frequency	41.8% (39.7, 44.0)	0.10			
Nuclear Cataract, Degree (0–7)	3.2 ± 1.1	0.04	0.05	0.18	0.01, 0.34
Cortical Cataract, Frequency	16.5% (14.9, 18.1)	0.05	0.04	0.48	-0.001, 0.97
Cortical Cataract, Degree (Percentage of lens Cortical Opacification)	5.0 ± 14.5	0.13			
Subcapsular Posterior Cataract	5.6% (4.6, 6.6)	0.49			
Subcapsular Posterior Cataract, Degree (Percentage of lens Cortical Opacification)	0.6 ± 4.3	0.07			
Glaucoma, Frequency, Total	5.4% (4.5, 6.3)	0.50			
Open-Angle Glaucoma, Frequency	3.4% (2.7, 4.1)	0.52			
Primary Angle-Closure Glaucoma	2.1% (1.5, 2.6)	0.33			
Pseudoexfoliation, Frequency	6.2% (5.2, 7.2)	0.56			
Age-Related Macular Degeneration, Frequency, Total	26.6% (24.9, 28.4)	0.71			
Age-Related Macular Degeneration as Ordinal Parameter	1804 / 196 / 437 / 17	0.36			
Age-Related Macular Degeneration, Early Stage, Frequency	8.0% (7.0, 9.0)	0.42			
Age-Related Macular Degeneration, Intermediate Stage, Frequency	17.9% (16.0, 19.0)	0.50			
Age-Related Macular Degeneration, Late Stage, Frequency	0.7% (0.0, 1.0)	0.06			
Age-Related Macular Degeneration, Late Stage; Geographic Atrophy, Frequency	0.3% (0.0, 0.1)	0.84			
Age-Related Macular Degeneration, Late Stage; Neovascular Stage, Frequency	1.3% (1.0, 2.0)	0.04	0.04	1.03	0.03, 2.03
Diabetic Retinopathy, Frequency	3.5% (2.9, 4.3)	0.003	0.06	1.33	0.47, 2.19
Retinal Vein Occlusion, Total, Frequency	2.9% (2.2, 3.5)	0.53			
Central Serous Choroidopathy, Frequency	0.2% (0.003, 0.04)	0.38			
Polypoidal Choroidal Vasculopathy, Frequency	4 / 2452 (0.2% (0.00, 0.00))	0.002	0.06	6.29	2.29, 10.28

Using step-wise multi-variant analysis, we first dropped, due to collinearity, the parameters of glucose (variance inflation factor (VIF): 2.85) and self-reported income (VIF: 2.08). Due to a lack of statistical significance, we then dropped step by step degree of nuclear cataract (*P* = 0.94), systolic blood pressure (*P* = 0.78), intake of aspirin (*P* = 0.72), axial length (*P* = 0.60), concentration of glycosylated hemoglobin (*P* = 0.41), presence of diabetes (*P* = 0.89), region of habitation (*P* = 0.16), presence of subcapsular posterior cataract (*P* = 0.22) and cortical cataract (*P* = 0.50), frequency of alcohol consumption (*P* = 0.22), presence of neovascular AMD (*P* = 0.73) and age (*P* = 0.09). In the resulting model, higher serum concentration of hs-CRP was significantly (regression coefficient r: 0.21) associated with a higher frequency of diabetic retinopathy (*P* = 0.007) and polypoidal choroidal vasculopathy (*P* = 0.002), higher body mass index (*P* = 0.001), and lower level of education (*P* = 0.04).

If in the model the presence of diabetic retinopathy was replaced by the level of diabetic retinopathy, higher serum concentrations of hs-CRP were associated with a higher ETDRS-defined level of diabetic retinopathy (*P* = 0.007). If we added the frequencies of ocular diseases to the model, the presence of glaucoma (*P* = 0.99), open-angle glaucoma (*P* = 0.80), angle-closure glaucoma (*P* = 0.67), pseudoexfoliation (*P* = 0.18), nuclear cataract (*P* = 0.30), cortical cataract (*P* = 0.15), subcapsular cataract (*P* = 0.59), retinal vein occlusions (*P* = 0.33), central serous choroidopathy (*P* = 0.44), early stage of AMD (*P* = 0.46), intermediate stage of AMD (*P* = 0.20) and late stage of AMD (*P* = 0.91) including geographic atrophy (*P* = 0.60) or neovascular AMD (*P* = 0.68) were not significantly associated with the serum concentration of hs-CRP.

If instead of the raw data of the hs-CRP concentration the logarithmically transformed data were taken as dependent variable, this logarithmically transformed value were significantly (regression coefficient r: 0.39) correlated with a higher frequency of polypoidal choroidal vasculopathy (*P* = 0.01; beta: 0.05; B: 0.55; 95%CI: 0.12, 0.98), lower concentration of high-density lipoproteins (*P*<0.001; beta: -0.20; B: -0.24; 95%CI: -0.29, -0.19), lower level of education (*P*<0.001; beta: -0.09; B: -0.04; 95%CI: -0.06, -0.02), and higher body mass index (*P*<0.001; beta: 0.24; B: 0.03; 95%CI: 0.02, 0.03), and higher frequency of diabetic retinopathy (*P* = 0.03; beta: 0.04; B: 0.12; 95%CI: 0.01, 0.22).

Taking the parameter of an increased hs-CRP serum concentration (>3mg/L) as independent variable in a binary regression analysis, a higher frequency of an increased hs-CRP level was associated with higher frequency of diabetic retinopathy (*P* = 0.04; OR: 1.81; 95%CI: 1.03, 3.17), lower concentrations of high-density lipoproteins (*P*<0.001; OR: 0ß.41; 95%CI: 0.28, 0.60), lower educational level (*P* = 0.002; OR: 0.80; 95%CI: 0.70, 0.92) and higher body mass index (*P*<0.001; OR: 1.14; 95%CI: 1.10, 1.17), while the presence of polypoidal choroidal vasculopathy was not significantly (*P* = 0.06; OR: 6.89; 95%CI: 0.92, 51.4) associated with an increased hs-CRP concentration. Cognitive function score was no longer significantly (*P* = 0.71; OR: 0.99; 95%CI: 0.95, 1.03) associated with the hs-CRP concentration.

## Discussion

In our population-based study on Chinese aged 50+ years, higher serum concentration of hs-CRP was significantly associated with a higher frequency and degree of diabetic retinopathy and higher frequency of polypoidal choroidal vasculopathy after adjusting for the systemic parameters of lower serum concentration of high-density lipoproteins level of education, higher body mass index, lower level of education and lower cognitive function score (Table 2). Other major ocular disorders, namely glaucoma including open-angle glaucoma and angle-closure glaucoma, pseudoexfoliation, nuclear, cortical or subcapsular cataract, retinal vein occlusions, central serous choroidopathy, early, intermediate or late stage of AMD including geographic atrophy, did not show a statistically significant association with hs-CRP serum concentrations. It suggests that these diseases, in contrast to diabetic retinopathy and polypoidal choroidal vasculopathy, were not markedly associated with a major systemic inflammatory component.

**Table 2:** Associations (multivariate analysis) between the serum concentration of hs-C-reactive protein and other systemic and ocular parameters in the Beijing Eye Study.

Parameter	*P*-Value	Standardized Regression Coefficient beta	Non-Standardized Regression Coefficient B	95% Confidence Interval	Variance Inflation Factor
Diabetic Retinopathy (ETDRS Level)	0.007	0.06	1.35	0.37, 2.22	1.01
Polypoidal Choroidal Vasculopathy	0.002	0.06	6.22	2.24, 10.2	1.00
Serum Concentration of High-Density Lipoproteins (mmol/L)	<0.001	-0.12	-1.31	-1.77, -0.85	1.15
Cognitive Score	0.01	-0.07	-0.08	-0.13, -0.02	1.56
Level of Education	0.035	-0.06	-0.22	-0.42, -0.02	1.62
Body Mass Index (kg/m^2^)	0.01	0.06	0.06	0.01, 0.11	1.18

The association between higher serum concentrations of CRP and higher frequency and level of diabetic retinopathy has also been addressed in previous investigations, which also included other than East-Asian populations [28–36]. To cite an example, in the study performed by Sosongko and associates on 224 diabetic patients, increasing concentrations of CRP levels were associated with the occurrence of vision-threatening diabetic retinopathy in a multivariate analysis [29]. The results of these studies agreed with a meta-analysis, in which higher CRP levels were associated with the presence and severity of diabetic retinopathy [32]. In contrast in the Singapore Malay Eye Study on patients with diabetes, higher CRP levels and higher body mass index were associated with a lower frequency of diabetic retinopathy in a multivariate analysis [33]. A similar finding was obtained in a community-based observational cohort study on Chinese men, but not on women [34]. The discrepancy between the studies might have been due to differences in the structure of the multivariate analysis, i.e., which parameters were included for adjustment. This may be important since the CRP concentration as well as the presence and severity of diabetic retinopathy depend on a multitude of parameters. Hitherto, the present study is largest one, and the one with a population-based recruitment of participants, which assessed associations between the serum concentration of CRP and diabetic retinopathy. The finding of increased CRP concentrations with DR is also in agreement with the notion of diabetic retinopathy as an inflammatory disease [5,6]. It may support the use of intravitreally applied steroids for the therapy of diabetic retinopathy.

A higher concentration of hs-CRP was also associated with an increased frequency of polypoidal choroidal vasculopathy (Table 2). It agrees with previous studies by Kikuchi and colleagues, Cheng and associates and others [37–39]. It suggests a systemic inflammatory component in the etiology of polypoidal choroidal vasculopathy.

In our study, higher hs-CRP concentrations were associated with a higher frequency of late AMD in univariate analysis, while in the multivariate analysis the association was no longer significant (Fig 1). The association between serum concentrations of CRP and presence and stage of AMD has been addressed in previous investigations [1–4,7–9,11,12]. To cite examples, Robman and colleagues reported that elevated CRP levels were associated with the presence of late AMD in a cross-sectional analysis, while the association between elevated CRP levels and AMD progression was statistically not significant [12]. In the Rotterdam Study, elevated serum concentrations of CRP increased the odds ratio of the population-attributable risk of complement factor H Y402H for the incidence and progression of AMD [8]. In contrast, Smith and colleagues examined 1727 patients with late AMD and 1153 controls and did not find a statistical significant correlation between these CRP variants and any type of late AMD [40]. The discrepancy between the studies may be due to various reasons including the structure of the multivariate analysis. Since parameters of systemic inflammation, such as the hs-CRP concentration, and the presence of AMD depend on a multitude of parameters, inter-dependencies between these parameters, if not adjusted for, may influence the final result of an analysis.

As in previous studies, higher CRP concentrations were associated with a reduced cognitive function in our investigation (Table 2) [41,42]. The results of our study with respect to associations between the serum concentration of CRP and other systemic parameters, such as higher body mass index, higher concentrations of high-density lipoproteins and lower level of education, confirm findings obtained in previous investigations.

When discussing the results, limitations of our study should be considered. First, CRP measurements were available for 2452 or 70.7% of the whole study population. The participants as compared with the individuals without measurements of hs-CRP were significantly younger, came significantly more often from the urban region than from the rural area, had a significantly higher proportion of women versus men and a significantly shorter axial length, and they were significantly less myopic. A selection artefact might thus have occurred. It was however not the goal of our study to report on the mean hs-CRP concentration in the adult Chinese population but to assess relationships between the hs-CRP concentration and ocular parameters, so that a potential selection bias might not have markedly influenced the results and conclusions of our study. Second, the associations between a higher CRP level and a higher frequency of diabetic retinopathy and of polypoidal choroidal vasculopathy were statistically significant, however, the standardized regression coefficients were relatively lower (beta = 0.06) as was the number of patients with polypoidal choroidal vasculopathy. One therefore has to take into account these relative weaknesses in the statistical evaluation when the clinical meaning of the findings is discussed.

In conclusion, in Chinese aged 50+ years, higher serum concentration of hs-CRP was significantly associated with a higher frequency and degree of diabetic retinopathy and higher frequency of polypoidal choroidal vasculopathy. Other major ocular disorders, namely glaucoma including open-angle glaucoma and angle-closure glaucoma, pseudoexfoliation, nuclear, cortical or subcapsular cataract, retinal vein occlusions, central serous choroidopathy, early, intermediate or late stage of AMD including geographic atrophy, did not show a statistically significant major association with hs-CRP serum concentrations. It suggests that these diseases, in contrast to diabetic retinopathy and polypoidal choroidal vasculopathy, were not markedly associated with a major systemic inflammatory component.

## Supporting information

S1 dataset The microdata are available in the datafile: “BES-BES2011-CreactiveProtein-15-2018-08-13-10-Datafile.sav”. (SAV)
